# Supplementary material for: Complexome profiling of the Chlamydomonas psb28 mutant reveals TEF5 as an early PSII assembly factor
Source: Plant Cell. 2025 Mar 17;37(6):koaf055. doi: 10.1093/plcell/koaf055 (PMC12164590; doi:10.1093/plcell/koaf055)
Supplement: koaf055_Supplementary_Data [file koaf055_supplementary_data.zip › psb28_interactive.zip!/plots/intensity/qualityPlots/peptides/TEF8_Cre03.g182150.t1.2.html]

### Cre03.g182150.t1.2

Trivial name: TEF8  
SeqCoverage [median]: 41.10  
Mass: 28.31 kDa  
  
 

| Sequence | MissedCleavage | Mass [Da] | wtIntensity | muIntensity | Score |
| --- | --- | --- | --- | --- | --- |
| ATRQDINKR | 2 | 1100.61 | 10194.00 | 27172.72 | 170.47 |
| DKGLLLVVTASK | 1 | 1242.75 | 2728.99 | 9045.12 | 70.62 |
| GLLLVVTASK | 0 | 999.63 | 18742.74 | 107458.32 | 156.51 |
| LKLLEIQTGYR | 1 | 1332.78 | 0.00 | 4022.06 | 53.57 |
| LLEIQTGYR | 0 | 1091.60 | 4559.03 | 14416.24 | 148.52 |
| LLGNPVPEAPVR | 0 | 1260.72 | 52095.37 | 176359.94 | 181.87 |
| LLGNPVPEAPVRNEQNR | 1 | 1902.01 | 111589.56 | 159206.59 | 165.34 |
| LLGNPVPEAPVRNEQNRER | 2 | 2187.15 | 14837.94 | 17747.63 | 151.04 |
| RLEFETDAFAFADK | 1 | 1658.79 | 1091.49 | 15903.39 | 148.18 |
| TYRTKEETEK | 2 | 1283.64 | 0.00 | 1394.62 | 106.16 |
| TYRTKEETEKSR | 3 | 1526.77 | 0.00 | 2826.69 | 148.89 |
| VEVVTVR | 0 | 800.48 | 6031.78 | 32038.34 | 94.26 |
| VLENWYPTAEAGK | 0 | 1476.72 | 1979.19 | 4090.19 | 123.51 |
| VLENWYPTAEAGKDK | 1 | 1719.85 | 6442.36 | 10129.11 | 105.14 |
| YNQTVVSAVER | 0 | 1264.64 | 14882.46 | 33387.42 | 273.44 |
|  |  |  | 245174.9 | 615198.4 |  |
